# Prognostic Accuracy of qSOFA and SIRS for Mortality in the Emergency Department: A Meta-Analysis and Systematic Review of Prospective Studies

**DOI:** 10.1155/2022/1802707

**Published:** 2022-05-05

**Authors:** Hailin Ruan, Dianshan Ke, Dalin Liao

**Affiliations:** ^**1**^ Department of Emergency Medicine, Liuzhou Workers' Hospital, The Fourth Affiliated Hospital of Guangxi Medical University, Liuzhou, Guangxi, China; ^**2**^ Department of Orthopedics, Fujian Provincial Hospital, Fuzhou, Fujian, China; ^**3**^ Department of Emergency, 900 Hospital of the Joint Logistics Team, Dongfang Hospital, Xiamen University, Fuzong Clinical College of Fujian Medical University, Fuzhou, Fujian, China

## Abstract

**Objective:**

This meta-analysis aimed to determine the prognostic performance of quick sequential organ failure assessment (qSOFA) score in comparison to systemic inflammatory response syndrome (SIRS) in predicting in-hospital mortality in the emergency department (ED) patients.

**Methods:**

Eligible studies comparing the performance of qSOFA and SIRS in predicting in-hospital death of ED patients were identified from searching PubMed, Embase, and Cochrane. Raw data were collected, and the pooled sensitivity and specificity were calculated for qSOFA and SIRS. The summary receiver operating curve was also plotted to calculate the area under the curve.

**Results:**

A total of 16 prospective studies with 35,756 patients and 2,285 deaths were included. The pooled sensitivity was 0.43 (95% CI: 0.32–0.54) and 0.8 (95% CI: 0.73–0.86) for qSOFA and SIRS, respectively. The pooled specificity was 0.89 (95% CI: 0.84–0.93) and 0.39 (95% CI: 0.3–0.5) for qSOFA and SIRS, respectively. The area under the summary receiver operating curve was 0.76 (95% CI: 0.72–0.8) and 0.67 (95% CI: 0.62–0.72) for qSOFA and SIRS, respectively. A significant heterogeneity was observed for both qSOFA and SIRS studies.

**Conclusion:**

The present meta-analysis suggested that qSOFA had a higher specificity but a lower sensitivity as compared with SIRS in predicting in-hospital mortality in the ED patients. qSOFA appeared to be a more concise and simple way to recognize patients at high risk for death. However, the use of SIRS in the ED cannot be completely replaced since the sensitivity of qSOFA was relatively lower.

## 1. Introduction

Sepsis is a leading cause of critical morbidity and mortality in the emergency department (ED) worldwide [1, 2], accounting for approximately 10% of intensive care unit (ICU) admission and 10–20% of in-hospital death [3–5]. However, recognizing patients with suspected infection who are at higher risk of sepsis and subsequent death in the ED setting is still a crucial challenge for clinicians. Therefore, an easy-to-use, fast, and accurate measure for predicting the prognosis of sepsis might be of great help for ED clinicians to determine whether more intensive monitoring and aggressive treatment should be applied to prevent adverse outcomes and ultimately in-hospital mortality. So far, a few scoring systems, such as systemic inflammatory response syndrome (SIRS) criteria, quick sequential organ failure assessment score (qSOFA), and National Early Warning Score (NEWS), have been devised for patients with suspected sepsis. These systems are considered as an effective tool for patients with critical illness, especially those in ICU [6]. However, in the ED setting, the performance and applicability of these scoring systems remain undetermined.

The first sepsis criterion, SIRS, was published in 1992 by the American College of Chest Physicians/Society of Critical Care Medicine consensus [7]. Accordingly, 4 SIRS criteria were defined as follows: tachypnea (respiratory rate more than 20 breaths per minute), tachycardia (heart rate more than 90 beats per minute), leukocytosis or leukopenia (leukocyte count more than 12,000 per *μ*L or less than 4000 per *μ*L, respectively), and fever or hypothermia (body temperature >38°C or <36°C, respectively). The consensus definition of sepsis required a defined infection and two or more SIRS criteria. Thereafter, in a number of studies, SIRS has been found to be of high sensitivity but unsatisfactory specificity for the diagnosis of sepsis and predicting in-hospital death [8]. In recent years, sepsis has been redefined by an international task force in The Third International Consensus Definitions for Sepsis and Septic Shock [9]. A new set of criteria, qSOFA, was adopted after the Sepsis-3 recommendations and was defined as follows: systolic arterial blood pressure no higher than 100 mmHg, respiratory rate more than 21 breaths per minute, and altered mental status. Increased qSOFA score was considered to be related with higher probability of mortality. The definition group stated that qSOFA might be a better indicator for in-hospital death as compared with SIRS and suggested using a qSOFA score of ≥2 to recognize patients at high risk for death due to infection, instead of a SIRS score of ≥2. However, the advantages and disadvantages of both traditional and new criteria in specific clinical settings, such as the ED, remain to be further clarified.

Since qSOFA was proposed, there have been emerging clinical investigations comparing the prognostic value of SIRS and qSOFA in predicting mortality of patients with suspected infection in the ED. Quite a few studies revealed a better performance of the new criteria. For instance, Freund et al. prospectively compared the predictive ability of qSOFA with the previous ones in the ED setting and observed a greater prognostic accuracy for in-hospital mortality of qSOFA than did either SIRS or severe sepsis [10]. The area under the receiver operating curve (AUROC) was 0.80 (95% CI: 0.74–0.85) for qSOFA versus 0.65 (95% CI: 0.59–0.70) for both SIRS and severe sepsis (*P* < 0.001) among 879 patients presenting to the ED with suspected infection [10]. The qSOFA criteria also showed a higher hazard ratio of 6.2 (95% CI: 3.8–10.3) versus 3.5 (95% CI: 2.2–5.5) for severe sepsis [10]. Similarly, in another recent prospective study of 2,045 patients with diagnosed or suspected infections in the ED, Abdullah et al. reported a higher 28-day mortality rate for qSOFA score ≥2 (17.8%; 95% CI: 12.4–24.3) as compared with SIRS criteria ≥2 (8.3%; 95% CI: 6.7–10.2) [11]. The AUROC for qSOFA was also higher than SIRS (0.63 vs. 0.52) [11]. As reported by Askim et al., qSOFA failed to identify at least 66% of the patients admitted to the ED with severe sepsis with a low sensitivity to predict 7-day and 30-day mortality [12]. Besides, a single-centre prospective study conducted by Graham et al. investigated the performance of a variety of scoring criteria in unselected ED patients [13]. The results showed a better prognostic value of SIRS criteria for prediction of 30-day mortality with a higher AUROC of 0.61 (95% CI: 0.58–0.64) than that of qSOFA criteria (0.56; 95% CI: 0.53–0.58) [13]. Thus, there is still significant controversy regarding the merits and shortcoming of qSOFA and SIRS.

Two years after qSOFA was proposed, Jiang et al. performed a head-to-head meta-analysis to compare qSOFA and SIRS criteria in predicting the mortality of infected patients admitted to the ED [14]. They included 8 studies with a total of 52,849 patients and observed that qSOFA score ≥2 indicated a higher probability of mortality in ED patients with infections than SIRS score ≥2 (risk ratio = 4.55 versus 2.75) [14]. However, Jiang et al. also stated that their meta-analysis included a relatively small number of studies (*n* = 8), and there were limited number of prospective studies (*n* = 4), which might not have enabled a complete evaluation of the predictive ability of qSOFA and SIRS [14]. Therefore, with the emerging clinical data on the comparison of qSOFA and SIRS in recent 3 years, we further performed this meta-analysis based on prospective studies. In the present meta-analysis, we aimed to compare the prognostic accuracy of qSOFA and SIRS for mortality in the emergency department including the diagnostic odds ratio (OR), sensitivity, and specificity as well as the area under the summary receiver operating curve (SROC).

## 2. Methods

### 2.1. Search Strategy and Selection Criteria

We searched all literature focusing on the performance of qSOFA and SIRS for the mortality of patients visiting ED through November 2021 in the PubMed, Embase, and Cochrane databases. The key words for literature search were as follows: for ED: emergency department, emergency, emergencies, urgent, and emergent; for qSOFA: qSOFA, Sequential Organ Failure Assessment, or Organ Failure Assessment; for SIRS: SIRS or systemic inflammatory response syndrome or inflammatory response syndrome.

**Figure 1:**
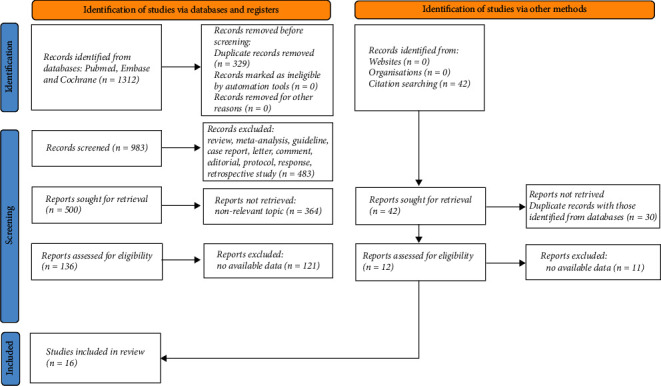
Flowchart of selection of studies.

The literature search was supplemented by checking the reference lists of the literature identified. The literature was managed by using EndNote (version X7). The protocol of this meta-analysis was registered in the International Prospective Register of Systematic Reviews (PROSPERO, registration ID: CRD42021289556). The eligibility of searched literature for inclusion was independently reviewed by two authors, and the divergence in the decision was issued via discussion with the other author. All human-based studies were considered eligible for inclusion if the following criteria were met: (1) prospective study; (2) the outcome was in-hospital death; (3) the data of sensitivity or specificity for predicting in-hospital death were reported for both qSOFA and SIRS. The literature was excluded if it was: (1) duplicate literature; (2) review, meta-analysis, or guideline; (3) case report, letter, comment, editorial, protocol, or reply; (4) with less than 20 patients; (5) basic research; (6) topic nonrelevant; (7) not in English; (8) without appropriate data (Figure 1). To fulfill the aim of the meta-analysis and obtain a homogeneous population, the participants of included studies should also fit the following criteria: (1) consecutively enrolled; (2) patients visiting the emergency department; (3) both qSOFA and SIRS were prospectively obtained at the emergency department; (4) repeat admissions during the study period should be excluded; (5) patients transferred from another hospital with pretreatment should be excluded; (6) age >16 years.

### 2.2. Data Extraction and Definitions

Raw data were collected by two authors independently. If there existed dissonance of extracted data by the two authors, a third author would preside over a discussion until consensus was achieved. The following data were collected: first author, time of publication, study location, size and age of population, participant selection, number of deaths, and sensitivity and specificity of qSOFA and SIRS for in-hospital death.

The primary outcome was in-hospital death, defined as patients visiting the ED and died with 28 days during treatment in hospital. If mortality was evaluated within both 28 days and other time-span, 28 days was the priority choice. If mortality was estimated only within other time-span such as 7 days, then it was also considered as appropriate data.

### 2.3. Data Synthesis and Statistical Analysis

The MIDAS module of the STATA software, version 12.0 (Stata Corporation, College Station, TX), and Meta-DiSc 1.4 (XI Cochrane Colloquium, Barcelona, Spain) were used to calculate the overall sensitivity, specificity, and SROC. The data were presented as the pooled estimates with 95% CIs. The heterogeneity was evaluated by the *Q* and *I*^2^ statistics. The posttest probability was also calculated and depicted through the MIDAS module of the STATA software based on Bayes' theorem. To estimate the possible sources of heterogeneity, subgroup analysis was performed according to the characteristics of included literature such as study location (Europe, Asia, or others), publication time (before 2020 or 2020 and after), and bias risk of articles (low risk, unclear, and high risk). A sensitivity analysis was also conducted by omitting one literature at each analysis.

### 2.4. Assessment of Quality of Studies and Publication Bias

The quality of study and publication bias were evaluated by RevMan version 5.3 via assessing the risk of bias (patient selection, index test, reference standard, and flow and timing) and applicability concerns (patient selection, index test, and reference standard).

The original dataset used for analysis is available in the Supplementary Materials.

## 3. Results

### 3.1. Search Results and Study Characteristics

The flowchart of literature selection is shown in Figure 1. A total of 1,312 and 42 literature were initially identified through database searching and citation searching, respectively. After removal of duplicates and screening by reviewing title, abstract, and full-text, a total of 16 prospective studies [10–13, 15–26] were eventually included.

The characteristics of included literature are listed in Table 1. All studies were published after 2016. Most of the studies were conducted in Europe (*n* = 7), while 5 in Asia and 4 in other regions. A total of 35,756 patients and 2,285 deaths among them were recorded. The majority of studies (*n* = 13) included individuals with suspected or confirmed infection, while the other studies (*n* = 3) included patients of other causes. The mean/median age of included population ranged from 30 to 83.6 years.

**Figure 2:**
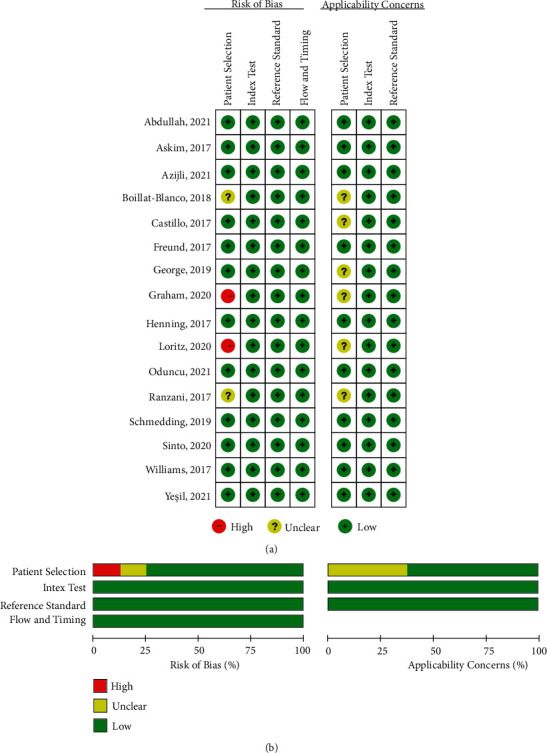
Risk of bias and applicability concerns of the included studies.

The quality was considered good for all included articles, probably due to the prospective study design (Figure 2). However, there was possible risk of patient selection bias in the studies by Boillat-Blanco et al. [16], Graham et al. [13], Loritz et al. [20], and Ranzani et al. [22]. The risk of bias was mainly because these studies included nonselective patients or patients of other causes, which were significantly different from the majority population with suspected or confirmed infection.

### 3.2. Performance of qSOFA and SIRS in Evaluating In-Hospital Mortality

**Figure 3:**
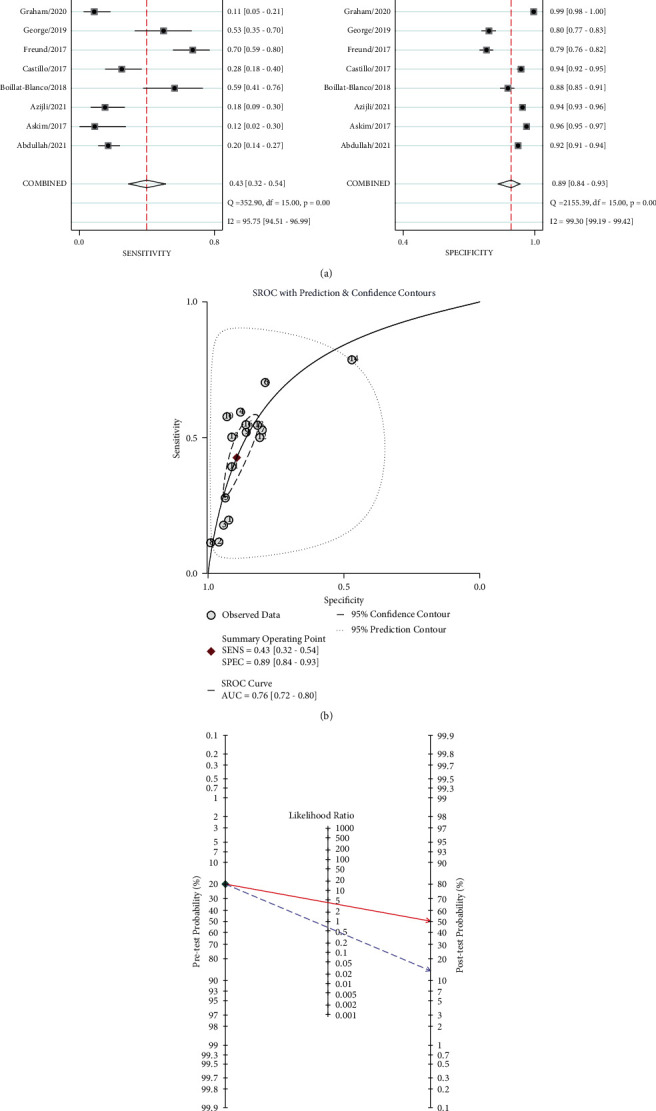
The forest plot of the (a) sensitivity and specificity of qSOFA, (b) the summary receiver operating curve of qSOFA, and (c) pre- and posttest probability of qSOFA for patients in the ED.

As shown in Figure 3(a), the sensitivity of qSOFA ranged from 0.11 to 0.79, resulting in a pooled sensitivity of 0.43 (95% CI: 0.32–0.54). A significant heterogeneity was identified with *I*^2^ = 95.8% (95% CI: 94.5%–97%). The specificity of qSOFA (Figure 3(a)) ranged from 0.47 to 0.99 with a pooled sensitivity of 0.89 (95% CI: 0.84–0.93). An obvious heterogeneity was also identified with *I*^2^ = 99.3% (95% CI: 99.2%–99.4%). Furthermore, the pooled SROC curve (Figure 3(b)) showed that the area under the curve (AUC) was 0.76 (95% CI: 0.72–0.8). The posttest probability was 50% for positive qSOFA (likelihood ratio = 4) and 14% for negative qSOFA (likelihood ratio = 0.64) (Figure 3(c)).

**Figure 4:**
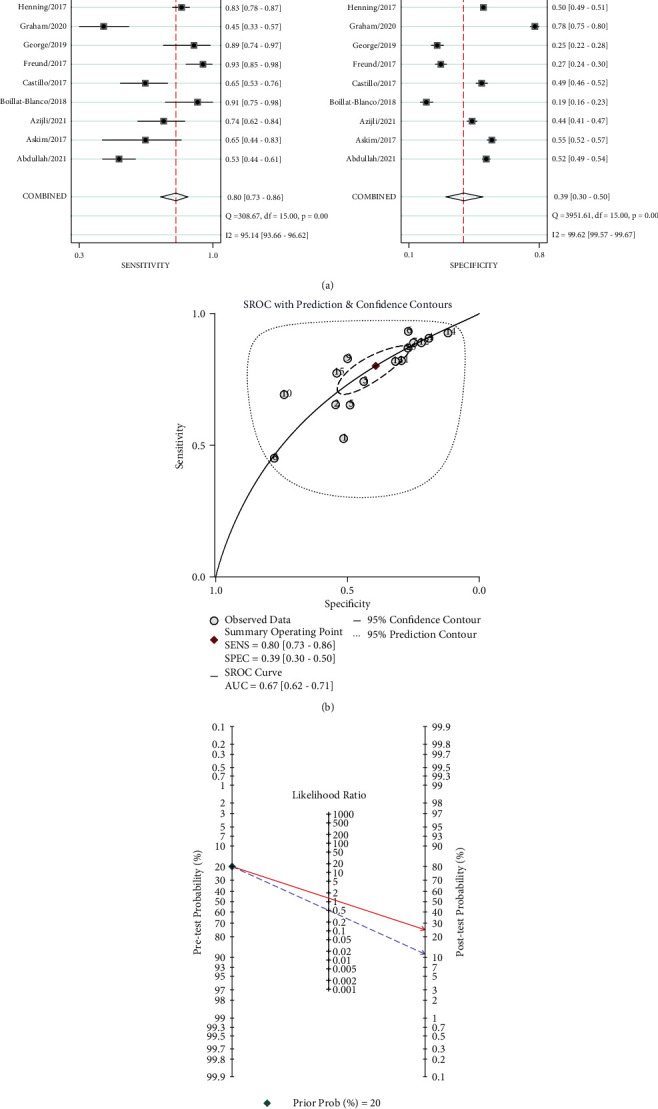
The forest plot of the (a) sensitivity and specificity of SIRS, (b) the summary receiver operating curve of SIRS, and (c) pre- and posttest probability of SIRS for patients in the ED.

As for SIRS, the sensitivity ranged from 0.45 to 0.93 and the pooled sensitivity was 0.8 (95% CI: 0.73–0.86) (Figure 4(a)). There was substantial heterogeneity with *I*^2^ = 95.1% (95% CI: 93.7–96.6). The specificity of SIRS ranged from 0.12 to 0.78, yielding an overall sensitivity of 0.39 (95% CI: 0.3–0.5). The heterogeneity was also significant from the specificity of SIRS with *I*^2^ = 99.6% (95% CI: 99.6–99.7) (Figure 4(a)). As shown by the pooled SROC curve (Figure 4(b)), the AUC for SIRS was 0.67 (95% CI: 0.62–0.72). The posttest probability was 25% for positive SIRS (likelihood ratio = 1) and 11% for negative SIRS (likelihood ratio = 0.51) (Figure 4(c)).

Taken together, qSOFA had relatively lower sensitivity but higher specificity as compared with SIRS. The performance of qSOFA in estimating in-hospital mortality was better than SIRS with a higher AUC.

### 3.3. Subgroup Analysis

A subgroup analysis restricted to different study locations, publication time, and bias risk of articles was performed (Table 2 and Supplementary Materials). In all of the subgroups, qSOFA still showed lower sensitivity but higher specificity than SIRS did. Only in the studies (*n* = 4) performed in the region other than Europe and Asia, the heterogeneity could be reduced to zero, while in all the other subgroups, the heterogeneity remained significant (*I*^2^ > 75%). Thus, the heterogeneity might mainly result from the different study locations.

### 3.4. Sensitivity Analysis of Sensitivity and Specificity for qSOFA and SIRS

The sensitivity of qSOFA varied from 0.4 to 0.46, and the sensitivity varied from 0.88 to 0.91 when omitting one study at each analysis (Table 3 and Supplementary Materials). On the other hand, the sensitivity of SIRS ranged from 0.79 to 0.82 and the sensitivity ranged from 0.37 to 0.42 when omitting one study at each analysis. This suggested that the results were relatively stable since no significant variation was observed when each of the studies was omitted.

## 4. Discussion

Early detection and intervention of sepsis have been paid increasing attention due to the rising incidence of sepsis-related death and health expenses in recent years. The incidence of sepsis is estimated to be 31.5 million according to the statistics by Fleischmann et al. in 2016 [2], and 5.3 million deaths among them were recorded. In the present meta-analysis, the SROC curve demonstrated that qSOFA had a better prognostic performance for in-hospital mortality in ED patients as compared with SIRS. Besides, qSOFA showed a higher specificity and positive likelihood ratio which also supported that it could be an effective tool for detecting infected patients at high risk of developing adverse outcomes. Thus, it might be necessary for clinicians in the ED to estimate the potential existence of organ dysfunction and consider ICU transfer for infected patients with qSOFA ≥2. The main results of this meta-analysis agreed with those of the early meta-analysis by Jiang et al. However, this meta-analysis included a larger number of only prospective studies. To our knowledge, this is the first meta-analysis and systematic review including only prospective studies comparing the prognostic value of the new sepsis definitions with the previous definitions in predicting in-hospital death in ED patients with suspected infections. As is known, the superiority of prospective study is that the detailed data of qSOFA and SIRS score are collected before the outcome of in-hospital death and therefore cannot be biased by the outcome. Thus, the results in this meta-analysis of prospective studies might be more accurate and further demonstrated the conclusion.

In certain circumstance, the sensitivity of diagnostic criteria is also important for the detection and mortality of sepsis, especially in the ED. As for the case of sepsis with high mortality, the potential life-threatening risk should be paid more attention than the cost for false positivity (unnecessary antibiotics or unnecessary hospitalizations). A test with high sensitivity is necessary for cases in which missing a potentially severe disease condition may lead to adverse clinical consequences. Thus, a diagnostic criterion with high sensitivity is also preferred in recognizing of sepsis and predicting in-hospital death [27]. In most previous studies [24–26] as well as in the present meta-analysis, SIRS showed a relatively higher sensitivity than qSOFA. Therefore, SIRS is also useful in initially screening patients with sepsis and is helpful for avoiding missed diagnosis. On the other hand, a highly specific test is also required since false-positive diagnosis may result in excessive burden on the patients. A highly sensitive bedside diagnostic tool can detect most of the patients with severe disease at an early stage and facilitate early intervention, while a highly specific diagnostic test can help clinicians in preventing overdiagnosis, overexamination, and overtreatment [28]. Thus, both SIRS and qSOFA criteria contribute to the identification of infected patients at high risk of serious outcomes in the ED. Besides, the qSOFA result could vary rapidly over a short time frame during in-hospital treatment, suggesting that a solitary calculation of qSOFA criteria on first visit might be insufficient. Future studies should investigate the relation of serial measurements of qSOFA and the adverse outcome, which might help more accurately determine the better time window for measuring qSOFA criteria during the ED stay.

The linear combination method of these score systems can simplify the bedside evaluation process, but the effect of the terms should not be as simple as 0 or 1. There are several ways to address the problem such as the ensemble modeling method and the nomogram, which can automatically take the nonlinearity effect of variables into analysis during the modeling process. These complex but more accurate model methods will be practicable as the electronic technology improves in the near future. Also, more studies are warranted to further investigate the prognostic effect of these terms for calculating risks.

There are several strengths of this meta-analysis. This meta-analysis only includes prospective studies, thus minimizing the potential risk of reporting bias and missing data which are frequently observed in many retrospective studies of sepsis. The prospective design of the included studies ensures that all patients visiting the ED are consecutively screened and included in the research. Furthermore, to the best of our knowledge, this is the largest meta-analysis of prospective studies comparing the performance of qSOFA and SIRS for patients with suspected infection in the ED setting. However, the heterogeneity is a limitation of this meta-analysis. The heterogeneity can be reduced in the subgroup of study location, suggesting that the heterogeneity mainly originates from the different regions of study populations. Notably, in the former meta-analysis by Jiang et al. [14], the heterogeneity was also significant in the combined population as well as in subgroups, yet the study location-associated heterogeneity was not evaluated in their analysis. Thus, further research should pay more attention to the variation of population characteristics in different geographical locations.

## 5. Conclusion

In summary, our analysis suggested that both qSOFA score and SIRS score were helpful risk stratification tools which are strongly associated with in-hospital death for patient in the ED with infections. qSOFA appears to be a more concise and effective index to detect patients at high risk for death. However, the sensitivity of qSOFA was relatively lower and the use of SIRS in the ED cannot be completely replaced.

## Figures and Tables

**Table 1:** Characteristics of included articles.

First author	Publication year	Study location	No. of patients	No. of deaths	Participant selection	Mean/median age (years)
Abdullah	2021	Denmark	2045	158	With suspected infection	73.2
Askim	2017	Norway	1535	26	With suspected infection	62
Azijli	2021	The Netherlands	1328	62	With suspected infection	62.6
Boillat-Blanco	2018	Tanzania	519	32	With fever	30
Castillo	2017	Spain	1071	72	Older patients with suspected infection	83.6
Freund	2017	France	879	74	With suspected infection	67
George	2019	USA	713	36	With pneumonia	61
Graham	2020	China	1253	71	None	72
Henning	2017	USA	7637	333	With suspected infection	56.9
Loritz	2020	Germany	1668	52	Patients of any nontraumatic cause	63
Oduncu	2021	Turkey	463	84	With suspected infection	63
Ranzani	2017	Spain	6024	442	With a clinical diagnosis of CAP	66
Schmedding	2019	Gabon	187	11	With an infectious diagnosis	38
Sinto	2020	Indonesia	1213	421	With suspected infection	51
Williams	2017	Australia	8871	327	With suspected infection	49
Yeşil	2021	Turkey	350	84	With suspected infection	63

CAP, community-acquired pneumonia.

**Table 2:** Subgroup analysis of sensitivity and specificity for qSOFA and SIRS.

Subgroup	No. of articles	qSOFA				SIRS			
		Sensitivity	*I* ^2^ (%)	Specificity	*I* ^2^ (%)	Sensitivity	*I* ^2^ (%)	Specificity	*I* ^2^ (%)
Study location									
Europe	7	0.34	95	0.91	99	0.75	97	0.45	99
Asia	5	0.48	98	0.91	99	0.81	97	0.38	99
Others	4	0.52	0	0.84	88	0.87	80	0.31	99
Publication time									
Before 2020	9	0.48	82	0.88	98	0.84	83	0.36	99
2020 and after	7	0.37	98	0.91	99	0.75	98	0.44	99
Bias risk of articles									
Low risk	12	0.44	96	0.88	99	0.81	93	0.37	99
Unclear and high risk	4	0.4	96	0.94	99	0.82	96	0.39	99

qSOFA, quick sequential organ failure assessment; SIRS, systemic inflammatory response syndrome.

**Table 3:** Sensitivity analysis of sensitivity and specificity for qSOFA and SIRS.

	qSOFA		SIRS	
Article omitted	Sensitivity	Specificity	Sensitivity	Specificity
Abdullah et al.	0.45	0.89	0.82	0.39
Askim et al.	0.45	0.89	0.81	0.38
Azijli et al.	0.45	0.89	0.80	0.39
Boillat-Blanco et al.	0.42	0.90	0.79	0.41
Castillo et al.	0.44	0.89	0.81	0.39
Freund et al.	0.41	0.90	0.79	0.40
George et al.	0.42	0.90	0.79	0.40
Graham et al.	0.46	0.88	0.82	0.37
Henning et al.	0.42	0.90	0.80	0.39
Loritz et al.	0.42	0.89	0.81	0.37
Oduncu et al.	0.43	0.89	0.80	0.40
Ranzani et al.	0.42	0.90	0.79	0.41
Schmedding et al.	0.42	0.90	0.80	0.40
Sinto et al.	0.40	0.91	0.79	0.42
Williams et al.	0.42	0.89	0.80	0.38
Yeşil et al.	0.42	0.90	0.80	0.40

qSOFA, quick sequential organ failure assessment; SIRS, systemic inflammatory response syndrome.

## Data Availability

The data supporting the findings of this study are included within the article and its supplementary materials.

## Supplementary Materials

Supplementary Materials (1) The .xlsx file “original database” contains all the raw data extracted from the included articles which are used for generating the results of this study. (2) The file folder “sensitivity analysis-forest plots” contains all the forest plots in the section of sensitivity analysis. The file with the name of author indicates the study that was omitted in each sensitivity analysis. (3) The file folder “subgroup analysis-forest plots” contains all the forest plots in the section of subgroup analysis. Each forest plot corresponds to the subgroup in Table 2. ()
