# Supplementary material for: Prognostic Accuracy of qSOFA and SIRS for Mortality in the Emergency Department: A Meta-Analysis and Systematic Review of Prospective Studies
Source: Emerg Med Int. 2022 May 5;2022:1802707. doi: 10.1155/2022/1802707 (PMC9098353; doi:10.1155/2022/1802707)
Supplement: Supplementary Materials — (1) The .xlsx file “original database” contains all the raw data extracted from the included articles which are used for generating the results of this study. (2) The file folder “sensitivity analysis-forest plots” contains all the forest plots in the section of sensitivity analysis. The file with the name of author indicates the study that was omitted in each sensitivity analysis. (3) The file folder “subgroup analysis-forest plots” contains all the forest plots in the section of subgroup analysis. Each forest plot corresponds to the subgroup in Table 2. () [file 1802707.f1.zip › 1802707.f1/sensitivity analysis-forest plots.zip!/sensitivity analysis-forest plots/Oduncu-qSOFA.pdf]

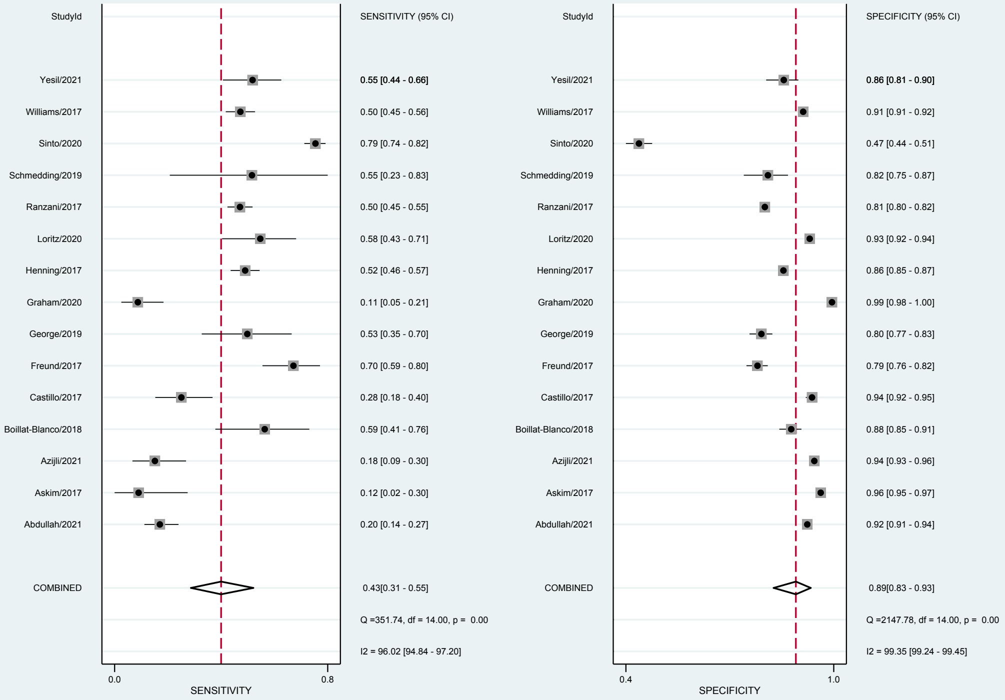
StudyId
SENSITIVITY (95% CI)
Yesil/2021
0.55 [0.44 - 0.66]
Williams/2017
0.50 [0.45 - 0.56]
Sinto/2020
0.79 [0.74 - 0.82]
Schmedding/2019
0.55 [0.23 - 0.83]
Ranzani/2017
0.50 [0.45 - 0.55]
Loritz/2020
0.58 [0.43 - 0.71]
Henning/2017
0.52 [0.46 - 0.57]
Graham/2020
0.11 [0.05 - 0.21]
George/2019
0.53 [0.35 - 0.70]
Freund/2017
0.70 [0.59 - 0.80]
Castillo/2017
0.28 [0.18 - 0.40]
Boillat-Blanco/2018
0.59 [0.41 - 0.76]
Azijli/2021
0.18 [0.09 - 0.30]
Askim/2017
0.12 [0.02 - 0.30]
Abdullah/2021
0.20 [0.14 - 0.27]
COMBINED
0.43[0.31 - 0.55]
Q =351.74, df = 14.00, p = 0.00
I2 = 96.02 [94.84 - 97.20]
0.0
0.8
SENSITIVITY
StudyId
SPECIFICITY (95% CI)
Yesil/2021
0.86 [0.81 - 0.90]
Williams/2017
0.91 [0.91 - 0.92]
Sinto/2020
0.47 [0.44 - 0.51]
Schmedding/2019
0.82 [0.75 - 0.87]
Ranzani/2017
0.81 [0.80 - 0.82]
Loritz/2020
0.93 [0.92 - 0.94]
Henning/2017
0.86 [0.85 - 0.87]
Graham/2020
0.99 [0.98 - 1.00]
George/2019
0.80 [0.77 - 0.83]
Freund/2017
0.79 [0.76 - 0.82]
Castillo/2017
0.94 [0.92 - 0.95]
Boillat-Blanco/2018
0.88 [0.85 - 0.91]
Azijli/2021
0.94 [0.93 - 0.96]
Askim/2017
0.96 [0.95 - 0.97]
Abdullah/2021
0.92 [0.91 - 0.94]
COMBINED
0.89[0.83 - 0.93]
Q =2147.78, df = 14.00, p = 0.00
I2 = 99.35 [99.24 - 99.45]
0.4
1.0
SPECIFICITY
